# The role of Sirt6 in osteoarthritis and its effect on macrophage polarization

**DOI:** 10.1080/21655979.2022.2059610

**Published:** 2022-04-21

**Authors:** Jinwei Chen, Sichun Chen, Dawei Cai, Qiugen Wang, Jian Qin

**Affiliations:** a Department of Orthopaedics Trauma Center, Shanghai General Hospital of Nanjing Medical University, Shanghai China; b Department of Orthopaedics, Sir Run Run Hospital, Nanjing Medical University, Nanjing, China

**Keywords:** Sirt6, osteoarthritis, synovium, macrophage, polarization

## Abstract

Osteoarthritis (OA), the commonest arthritis type, features irreversible cartilage loss and synovitis. It was reported that macrophages have an important function in synovial inflammation, and our team revealed that the amounts of Sirt6, a nicotinamide adenine dinucleotide (NAD)^+^-dependent histone deacetylase, decrease during synovial inflammation and osteoarthritis. This work aimed to examine the anti-inflammatory properties of Sirt6 in synovial inflammation. Firstly, we compared Sirt6 amounts in acute meniscus injury and OA human knee synovial tissue samples by immunofluorescence and immunoblot. Secondly, Sirt6ʹs suppressive effects on inflammatory markers and macrophage polarization were evaluated. Finally, OA mice were histologically evaluated, and serum inflammatory factors were detected for assessing the impact of Sirt6 overexpression on the mouse synovium. We found significantly lower interleukin-4 (IL-4) amounts and M2 polarization in OA patients compared with control individuals. The expression of Sirt6 was lower in RAW264.7 cells of the lipopolysaccharides (LPS) + interferon-gamma (IFN-γ) group compared with the phosphate buffer saline (PBS) group, but higher than in the IL-4 group. The polarization of macrophages affected Sirt6 expression, which was reduced and elevated in M1 and M2 macrophages, respectively. Sirt6 inhibition could promote the release of proinflammatory cytokines by macrophages in the synovial membrane, induce M1 polarization in macrophages and inhibit M2 polarization *in vitro*, and Sirt6 overexpression alleviated osteoarthritis *in vivo*. These data strongly suggested that Sirt6 could inhibit synovial inflammation. Thus, this study provides a novel therapeutic target in osteoarthritis.

## Graphical abstract

**Figure:**
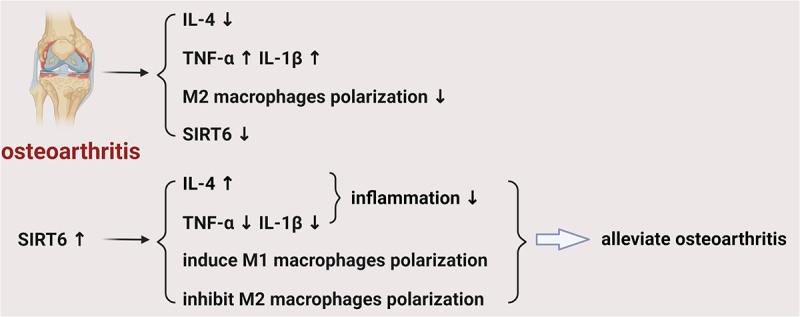

## 1. Introduction

Osteoarthritis (OA), the commonest joint pathology, can occur in almost all moving joints, especially the knee joint. It is a complex disease involving the articular cartilage, subchondral bone, synovium, ligaments, joint capsule and muscles around the joint [1–3]. As a common disease causing disability, it is estimated that about 250 million individuals are affected by OA around the world; in addition, with the coming of the ‘global aging era’, huge medical, economic and financial burdens have brought new challenges to individuals, health care systems and the society [4,5]. Joint damage may result in cartilage corrosion defect, osteophyte formation, subchondral bone sclerosis, synovial hypertrophy and hyperplasia. Clinically, the initial manifestations are often joint pain, swelling and limited movement, which progressively lead to the deformity and function loss of the whole joint [6–8].

Many studies have confirmed inflammation occurrence in the synovium of OA patients, with the severity of synovitis closely related to OA severity and disease progression [9,10]. Moreover, it was reported that synovitis occurs prior to articular cartilage destruction, and is the precursor of OA development [11]. In the process of OA, inflammatory cytokines secreted by chondrocytes and synovial macrophages, including interleukin-1 beta (IL-1β) and tumor necrosis factor-α (TNF-α), increase the expression of degradation factors such as matrix metalloproteinase 3 (MMP3) and matrix metalloproteinase 13 (MMP13) in the joint, thereby promoting the degradation of cartilage matrix, inducing apoptosis of articular cartilage cells and eventually causing cartilage damage [12]. The primary role of macrophages is to maintain tissue homeostasis and protect the host from infection. Although primarily considered to be critical components of innate immunity, macrophages are capable of bridging and instructing the response of the adaptive immune system via various secretory mediators [13]. Macrophages are the main inflammatory cells in synovial tissue. Pro-inflammatory factors and cartilage matrix degrading enzymes secreted by macrophages are important factors that aggravate OA and accelerate joint degeneration [14,15]. Therefore, understanding the roles of synovial macrophages in OA development and progression is of prime importance. Sirt6 is an important nicotinamide adenine dinucleotide (NAD)^+^-dependent histone deacetylase. Belonging to the Sirtuins family, Sirt6 affects biological processes, e.g., by delaying aging, inhibiting inflammation and stabilizing the genome [16]. Kanfi et al. demonstrated transgenic male mice overexpressing Sirt6 have significantly longer life span compared with wild type counterparts [17]. Lee and colleagues revealed Sirt6 overexpression significantly inhibits TNF-α induced inflammatory responses in human rheumatoid fibroblast-like synovial cells. In addition, they found that Sirt6 could generally reduce the number and activity of osteoclasts and inhibit cartilage damage in the collagen-induced arthritis mice model [18]. Wu et al. found that Sirt6 is obviously suppressed in articular chondrocytes from OA cases compared with healthy individuals. Sirt6 overexpression prevents interleukin-1β-induced chondrocyte senescence and osteoarthritic changes [19]. However, the function of Sirt6 in OA synovial macrophages is unclear.

In the present study, we hypothesized that Sirt6 amounts in synovial macrophages are closely related to OA. To validate our hypothesis, we examined the expression levels of Sirt6 and inflammatory factors in OA synovium. Then, we knocked down Sirt6 in RAW264.7 cells and overexpressed Sirt6 in OA mice to evaluate Sirt6 function in OA development through cellular and animal models.

## 2. Material and methods

### 2.1. Preparation of reagents, human synovial specimens and cells

High-glucose Dulbecco’s Modified Eagle Medium (HDMEM, Gibco, USA), Penicillin, streptomycin and fetal bovine serum (FBS) were provided by Gibco BRL(Grand Island, NY, USA). Primary antibodies targeting Sirt6, CD86, CD206, TNF-α, IL-1β, interleukin-4 (IL-4) and β-actin were used in this study, as well as Alexa-Fluor-488-conjugated and goat anti-rabbit IgG secondary antibodies. Anti-Sirt6, anti-TNF-α, anti-IL-1β, anti-IL-4 and anti-β-actin antibodies were provided by Abcam (Cambridge, UK). Anti-CD86 and anti-CD206 antibodies were acquired from Santa Cruz Biotechnology (Santa Cruz, CA). Alexa-Fluor-488-conjugated goat anti-rabbit IgG secondary antibodies were purchased from Jackson ImmunoResearch (West Grove, PA, USA). Recombinant human and mouse TNF-α, IL-1β and IL-4 were provided by R&D Systems (Minneapolis, MN). Lipopolysaccharides (LPS) and Recombinant Murine interferon-gamma (IFN-γ) were obtained from Sigma-Aldrich (USA). Primer synthesis was performed by Shanghai Ruibo Bioengineering (Shanghai, China).

OA synovial specimens were obtained from 8 patients undergoing knee replacement surgery (6 females and 2 males; ages of 64 to 76 years). The Kellgren-Lawrence score of knee joint X-ray imaging in OA group was from III to IV grade. Control human synovial specimens were obtained from patients undergoing arthroscopic surgery for acute meniscus tears (6 females and 2 males; ages of 28 to 46 years). The Kellgren-Lawrence score of knee joint X-ray imaging in control group was from 0 to I grade. The synovial tissues collected were fixed in 4% paraformaldehyde (PFA) solution.

To mimic the characteristics of OA in vitro, a cellular model was established in RAW264.7 cells, the murine macrophage cell line. RAW264.7 cells were provided by Shanghai Siding Biotechnology and underwent seeding into culture flasks at 3x10^5^/ml and culture in a 5% CO_2_ incubator at 37°C in HDMEM containing 10% FBS. Cell passage 1 was carried out at 70–80% confluence. RAW264.7 cells were administered LPS (1 μg/ml) and IFN-γ (20 ng/ml) for 24 h for M1 differentiation, while IL-4 (20 ng/ml) was used to stimulate RAW264.7 cell differentiation into the M2 type.

### 2.2. Histopathological analysis, immunohistochemistry (IHC) and immunofiuorescence

After embedding, specimens were sectioned at 5 µm in the sagittal plane, followed by hematoxylin and eosin (H&E) staining for histology. In order to evaluate the expression of Sirt6 in synovial tissue, the sections underwent immunohistochemical staining with anti-Sirt6 primary (overnight) and secondary (2 h at ambient) antibodies. Then, DAB (Zsbio, Beijing, China) was utilized for development, and counterstaining used hematoxylin. To evaluate Sirt6 staining and M2 macrophages in synovial cells, sections underwent three phosphate buffer saline (PBS) washes. At ambient, cell fixation was carried out with 4% PFA for 15 minutes. Next, the cells were covered with 5% protease-free BSA (Sigma-Aldrich, USA) at ambient for 1 h, followed by incubation with primary antibodies against CD206 (1:200) and Sirt6 (1:200) at 4°C overnight. After incubation with fluorescein-linked goat anti-rabbit IgG (1:500) at ambient (1 h), the cells underwent three PBS washes and 4’,6-diamidino-2-phenylindole (DAPI; Invitrogen, USA) counterstaining. Images were captured under a Zeiss LSM510 Meta laser-scanning confocal microscope (Carl Zeiss, Thornwood, NY). ImageJ 2.1 (Bethesda, MD, USA) was utilized to quantify the average optical density (AOD) values of the stained area. Red-stained areas were selected as the uniform standard for evaluating all images. Each image was analyzed to obtain the integrated optical density (IOD) and the area of the pixel (AREA) of the tissue. Finally, the AOD (AOD = IOD/AREA) was determined. Using the image scale (50 μm) as the standard, the cartilage thickness (millimeters) at 5 positions was measured for each image, and the average value was obtained.

### 2.3. Immunoblot

Total protein extraction from synovial tissue specimens or RAW264.7 cells was performed after lysis with sample buffer, followed by a 10-min centrifugation at 12,000 rpm and 4°C. Then, 40–50 µg total protein was resolved by sodium dodecyl sulfate-polyacrylamide gel electrophoresis (SDS-PAGE), followed by electro-transfer onto polyvinylidene fluoride (PVDF) membranes (Bio-Rad, USA). Blocking was carried out for 2 h with 5% skimmed milk, and membranes underwent a 2-h incubation with primary antibodies, as directed by the respective manufacturers overnight at 4°C; control membranes were incubated with anti-β-actin (1:1000) primary antibodies. Next, appropriate secondary antibodies were added at ambient for 2 h. After washing the blots three times with Tris-Buffered Saline and Tween (TBST), the electrochemiluminescence (ECL) reagent (Invitrogen, USA) was utilized for detection. Finally, Image Lab 3.0 (Bio-Rad) was used for the quantitation of immunoreactive bands.

### 2.4. Chemokine and cytokine analyses

For the determination of cytokine levels in OA and normal human serum specimens, pro-inflammatory (IL-1β and TNF-α) and anti-inflammatory (IL-4) cytokines were quantitated with high-sensitivity ELISA kits (R&D Systems), as directed by the manufacturer. In order to assess the levels of cytokines in mice, serum samples were examined on the fiftieth day.

### 2.5. Flow cytometry analysis

After indicated lentivirus injection, RAW264.7 cells were detached with 0.25% trypsin-EDTA (Gibco) for 20–30 s and centrifuged at 1000 rpm for 5 min. After the removal of supernatant, cells were washed twice by PBS, and then collected and resuspended in PBS. Cell surface markers, CD86-FITC and CD206-FITC, were used to label the cells on ice for 30 min in the dark. Cells were washed twice and resuspended in 200 μL of PBS before analysis. All antibodies were purchased from BD Biosciences. Flow cytometry was performed with a flow cytometer (Beckman Coulter).

### 2.6. Lentivirus transfection

The Lenti-X HTX Packaging system (Clontech; Mountain View, CA) was utilized, and a plasmid harboring Sirt6 (pCMV-SPORT6-Sirt6) was manufactured by Open Biosystems (Lafayette, CO). Sirt6 gene silencing by a lentiviral shRNA (Origene, Rockville, MD) was achieved by the Lenti-X shRNA Expression system. Sirt6 overexpression (Lenti-Sirt6) and the control Lenti-NC was also provided by Open Biosystems. The inserted fragment was amplified by PCR and connected to the multiple cloning site of the pLVX vector. The 293 T cells were co-transfected with the mixture of the lenti-X vector and lenti-X HTX with the Xfect transfection reagent (Clontech) to prepare recombinant lentiviruses. Totally 48 h after transfection, cell culture supernatants were obtained and treated with a 0.45 μm filter. RAW264.7 macrophages (30–50% confluent) underwent transfection with Lenti-Sirt6-siRNA and Lenti-NC-siRNA at a multiplicity of infection (MOI) of 200. More than 95% of cells survived after 12 h, which was followed by medium change. After three days, transfected cells underwent passaging for subsequent assays. Transfection efficacies were measured by Western blotting and qPCR.

### 2.7. RNA isolation and real-time PCR

Total RNA isolation used Trizol (Invitrogen) as directed by the manufacturer. Reverse transcription from 1 μg of RNA was carried out with One Step RT-PCR Kit (MBI Fermentas, Germany). Quantitative real-time PCR (qPCR) was carried out with the iQTM SYBR Green supermix PCR kit with the iCycler apparatus system (Bio-Rad, USA). The following primers were used: Sirt6, Forward 5’-GCAGTCTTCCAGTGTGGTGT-3’ and Reverse 5’-CCATGGTCCAGACTCCGT-3’; TNF-α, Forward 5’-ATGTCTCAGCCTCTTCTCATTC-3’ and Reverse 5’-GCTTGTCACTCGAATTTTGAGA-3’; IL-1β, Forward 5’- TCGCAGCAGCACATCAACAAGAG-3’ and Reverse 5’-TGCTCATGTCCTCATCCTGGAAGG-3’; IL-4, Forward 5’-GCTATTGATGGGTCTCACCC-3’ and Reverse 5’-CAGGACGTCAAGGTACAGGA-3’; GAPDH, Forward 5’-GGCACAGTCAAGGCTGAGAATG-3’ and Reverse 5’- ATGGTGGTGAAGACGCCAGTA-3’. GAPDH was utilized for normalization.

### 2.8. Animal OA models and histological assessment

Eight-week C57BL/6 male wild-type (WT) mice were provided by the Animal Center of Nanjing Medical University (Nanjing, China), and assigned to the Ad-LacZ+OA and Ad-Sirt6+ OA groups (n = 5). The two groups underwent medial collateral ligament transection and medial meniscectomy for inducing surgical OA. If the degenerative changes in articular cartilage of mice were observed, the OA model was successfully induced. Tail vein injection of 10 μL of Lenti-Sirt6 was performed at 0, 15, 30 and 45 days post-OA modeling. Control mice were administered 10 μL of Lenti-NC while the experimental group received Lenti-Sirt6. Mice in both groups were sacrificed at 8 weeks post-OA induction. Knee joints underwent dissection for histological assessment. Immunoblot and qPCR were utilized for detecting Sirt6 in synovial tissue of the mouse knee joint. Serum pro-inflammatory (TNF-α and IL-1β) and anti-inflammatory (IL-4) factors in mice were analyzed by ELISA at 50 days post-OA surgery. After mice were sacrificed, the knee joint was harvested 8 weeks postoperatively. Specimen fixation in 4% PFA was carried out for 24 h, followed by decalcification in neutral 10% EDTA for 28 days. After dehydration and paraffin embedding, tissue specimens were frozen and sectioned into 5 μm in the sagittal plane. Sections (5 µm) were stained with hematoxylin and eosin (H&E) or safranin O–fast green (S–O) for light microscopy. Articular cartilage specimens were evaluated by the Osteoarthritis Research Society International (OARSI) histological scoring system [20].

### 2.9. Statistical analysis

Data are mean ± standard deviation (SD) from two assays performed independently. Statistical significance was assessed by Student’s t-test with SPSS v19.0 (SPSS, Armonk, NY, USA). P < 0.05 indicates statistical significance.

## 3. Results

### 3.1. Sirt6 amounts in acute meniscus injury and OA human knee synovial tissue

**Figure 1.**
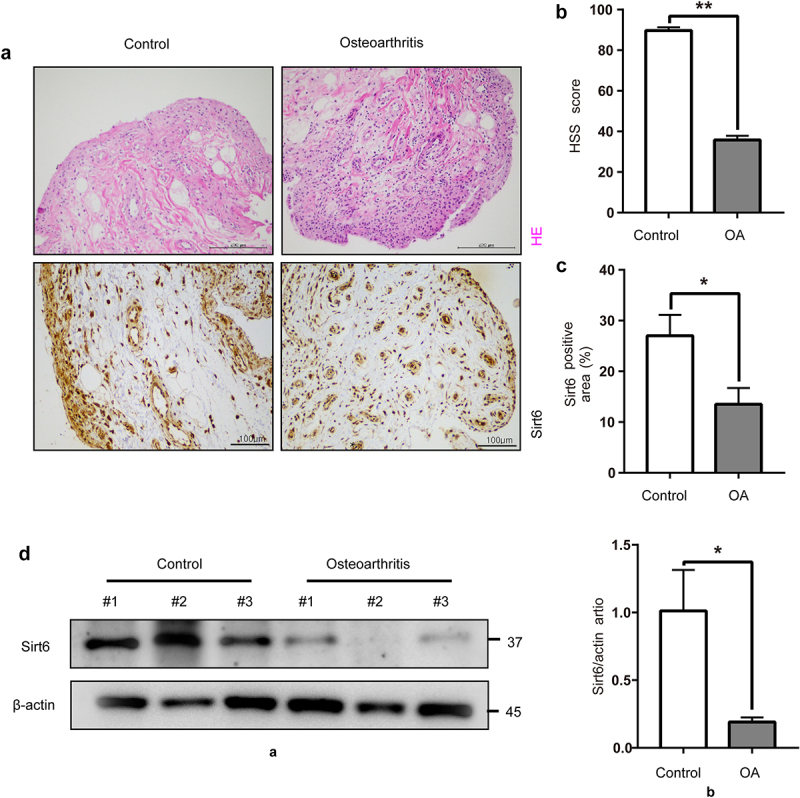
**Sirt6 amounts in knee synovial tissue specimens from acute meniscus injury individuals and OA cases**. (a). Representative images of H&E staining (Scale bar, 200 µm) and immunohistochemistry of Sirt6 (Scale bar, 100 µm) in synovial tissue specimens from acute meniscus injury individuals and OA cases. (b). Quantification of HSS scores for the knee joint in control (n = 8) and OA (n = 8) individuals. **P < 0.01 by Student’s t-test. (c). Quantitation of Sirt6-positive macrophages (proportion of total macrophages) in synovial tissues from control (n = 8) and OA (n = 8) individuals. *P < 0.05 by Student’s t-test. (d). Sirt6 protein amounts in synovial tissues from acute meniscus injury individuals and OA cases (n = 3, *P < 0.05).

For assessing the associated of Sirt6 amounts with OA, we observed acute meniscus injury and OA human knee joint synovial tissues by H&E staining. We found that synovial lining layer proliferation and inflammatory cell infiltration were more severe in OA compared with control synovial tissues (Figure 1a). HSS scores of the knee joint were reduced in the OA group comparison in the control group (Figure 1b). We first detected Sirt6 levels in knee joint synovial tissue of acute meniscus injury and OA human by immunohistochemistry for studying Sirt6ʹs in OA development. We found Sirt6 amounts were reduced in the knee synovial tissues of OA compared with control ones (Figure 1a,c). We further assessed differences in Sirt6 protein amounts between the two groups by immunoblot. Comparable findings were also obtained, Sirt6 protein amounts in the synovium of OA cases were markedly reduced compared with those of the control group (Figure 1d). We performed IF staining in OA and control synovial tissues. The results showed that CD206 and Sirt6 fluorescent intensity decreased in OA synovial tissues (Supplementary Fig. 1A), suggesting that Sirt6 presented high expression in macrophage M2 subtype in the OA synovial tissues.

### 3.2. Serum anti-inflammatory factor levels and synovial M2 macrophages are decreased in OA patients

**Figure 2.**
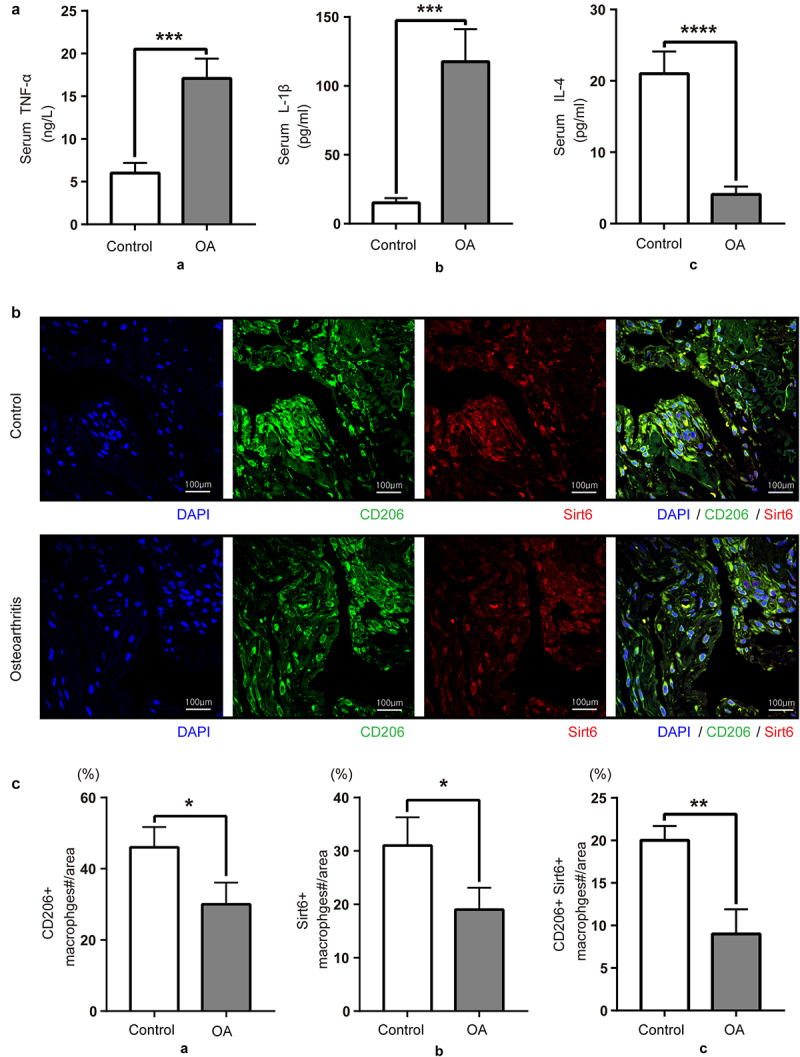
**Serum anti-inflammatory factors and synovial M2 macrophages are decreased in OA patients**. (a). TNF-α, IL-1β and IL-4 amounts in control and OA serum samples, assessed by ELISA. Data are mean ± SD. Compared with control patients, OA patients had elevated amounts of pro-inflammatory cytokines (TNF-α and IL-1β; ***P < 0.01, n = 8), and reduced levels of the anti-inflammatory factor IL-4 (****P < 0.01, n = 8). (b). Sirt6 and CD206 were assessed by immunofluorescence and DAPI counter staining (magnification, ×400; scale bar, 100 μm; nuclei, blue; CD206, green; Sirt6, red). (c). Quantitation of CD206 and Sirt6-positive macrophages relative to total macrophages with Image J (n = 8). *P < 0.05, **P < 0.01 by Student’s t-test.

We first examined inflammation and synovial macrophage phenotypes in acute meniscus injury individuals and OA cases to explore the function of synovial macrophages in OA development. Compared with control patients, serum TNF-α and IL-1β amounts were elevated in OA patients, while the levels of the anti-inflammatory factor IL-4 were decreased (Figure 2a). As depicted in Figure 2b-c, the fluorescence intensities of Sirt6 and the M2-like macrophage marker CD206 decreased in knee synovial tissue specimens from OA cases while monocyte-macrophage maker CD68 showed no significant change versus acute meniscus injury individuals. In brief, the above results demonstrated that compared with control patients, the amounts of Sirt6, the anti-inflammatory cytokine IL-4, and M2 macrophages in OA patients decreased, while those of the pro-inflammatory cytokines TNF-α and IL-1β were elevated.

### 3.3. Sirt6 inhibition induces RAW264.7 macrophages to release pro-inflammatory cytokines

**Figure 3.**
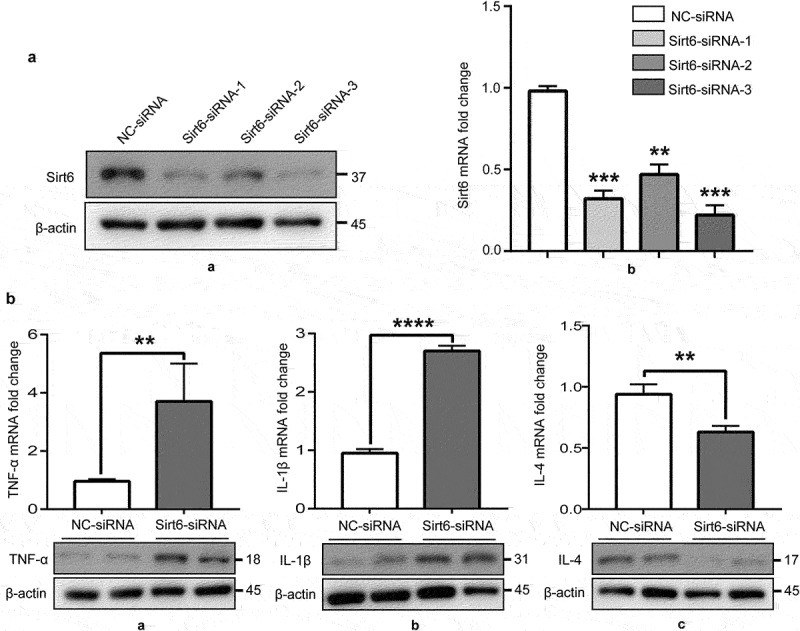
**Sirt6 inhibition promotes RAW264.7 macrophages to release pro-inflammatory cytokines**. (a). The protein and mRNA expression levels of Sirt6 were reduced upon Sirt6-siRNA transfection in RAW264.7 cells as examined by immunoblot and qPCR, respectively. (b). TNF-α, IL-1β and IL-4 protein and mRNA amounts, examined by immunoblot and qPCR, respectively.

To further examine whether Sirt6 exerts anti-inflammatory effects on synovial macrophages, RNAi targeting Sirt6 was introduced into RAW264.7 cells, divided into two groups: negative control-siRNA (NC-siRNA) and Sirt6-siRNA groups. The protein and mRNA amounts of Sirt6 were starkly reduced upon Sirt6 siRNA transfection in RAW264.7 cells (Figure 3a). Subsequently, we examined TNF-α, IL-1β, and IL-4 protein and mRNA amounts by immunoblot and qPCR, respectively. Sirt6 Inhibition induced RAW264.7 cells to release TNF-α and IL-1β, while reducing IL-4 levels (Figure 3b).

### 3.4. RAW264.7ʹs polarization state affects Sirt6 expression, and Sirt6 inhibition induces M1 polarization and inhibits M2 polarization in macrophages

**Figure 4.**
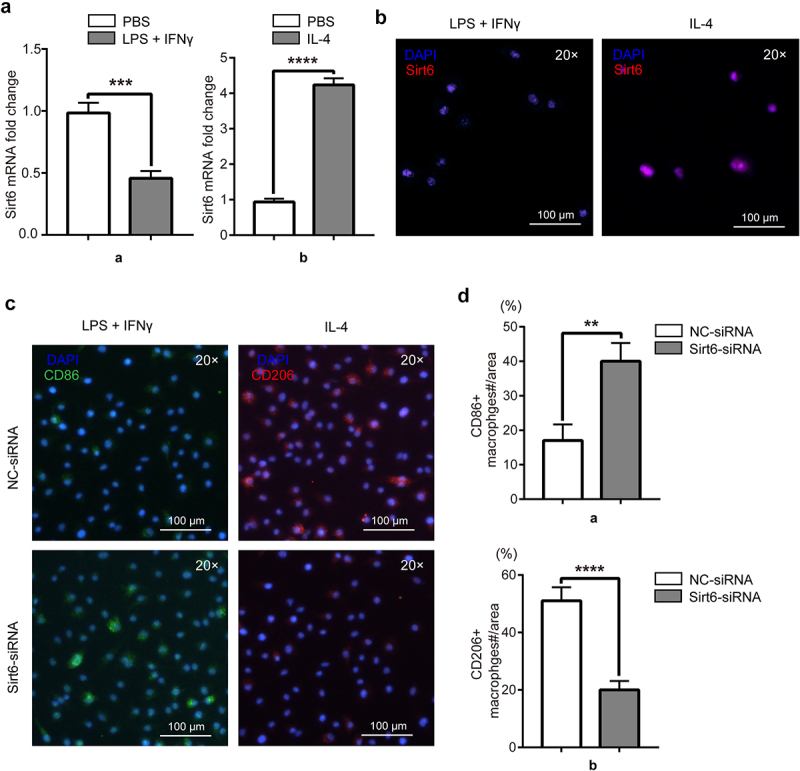
**RAW264.7 polarization state affects Sirt6 expression, and Sirt6 inhibition induces M1 polarization and inhibits M2 polarization in macrophages**. (a). The mRNA levels of Sirt6 in RAW264.7 cells stimulated as above were detected by qPCR. (b). Representative immunofluorescent images of Sirt6 in RAW264.7 cells of the LPS + IFN-γ and IL-4 groups (magnification, ×200; scale bar, 100 μm). (c). CD86 and CD206 were detected by immunofluorescence and DAPI counterstaining (magnification, ×200; scale bar, 100 μm). (d). Quantitative analysis of CD86 and CD206-positive macrophages relative to total macrophages with Image J. **P < 0.01, by Student’s t-test.

In order to examine the relationship between different phenotypes of macrophages and Sirt6, RAW264.7 cells were induced by LPS (1 μg/ml) and IFN-γ (20 ng/ml) to differentiate into M1 macrophages in the experimental group, and PBS was used in the control group. Similarly, RAW264.7 cells were induced by IL-4 (20 ng/ml) to differentiate into M2 macrophages in the experimental group. Sirt6 was detected by immunofluorescence and qPCR, and lower amounts were obtained in RAW264.7 cells administered LPS and IFN-γ compared with the PBS group. Meanwhile, Sirt6 expression was higher in RAW264.7 cells administered IL-4 versus the PBS group (Figure 4a-b). To examine Sirt6ʹs function in macrophage polarization, RAW264.7 cells stimulated with LPS and IFN-γ were assigned to the negative control-siRNA (NC-siRNA) and Sirt6-siRNA groups. The results showed that the fluorescence intensity of the M1-like macrophage marker CD86 was elevated in the Sirt6-siRNA group compared with the NC-siRNA group. RAW264.7 cells administered IL-4 were also divided into similar groups. The results showed that the fluorescence intensity of the M2-like macrophage marker CD206 was reduced in the Sirt6-siRNA group versus the NC-siRNA group (Figure 4c-d). Additionally, we performed WB to detect phenotypic changes of macrophages under Sirt6 knockdown or overexpression. The results showed that CD86 expression increased while CD206 expression decreased under Sirt6 knockdown, and the opposite results were observed under Sirt6 overexpression (Supplementary Fig. 1B). Similarly, flow cytometry analysis to CD86 and CD206 showed that CD86 was highly expressed while CD206 was lowly expressed in Sirt6-siRNA-tansfected RAW264.7 cells, and the opposite trend showed in the Ad-Sirt6 group (Supplementary Fig. 1C).

### 3.5. Sirt6 overexpression inhibits inflammation and alleviates mouse OA

**Figure 5.**
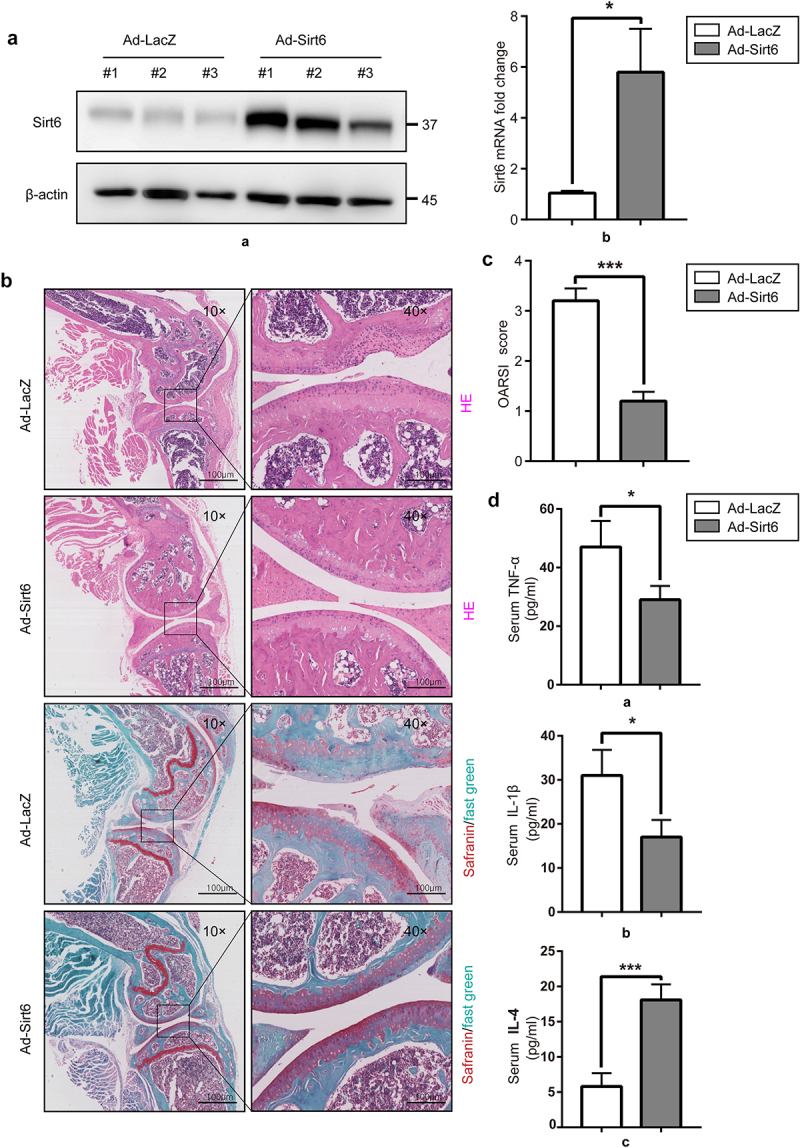
**Sirt6 overexpression alleviates osteoarthritis and inhibits inflammation in mice**. (a). The protein and mRNA levels of Sirt6 in synovial tissue samples from the knee joint in Ad-LacZ + OA and Ad-Sirt6 + OA mice were examined by immunoblot and qPCR, respectively (*P < 0.05, n = 5). (b). H&E and safranin O staining of the knee joint in the Ad-LacZ + OA and Ad-Sirt6 + OA groups at 8 weeks post-operation (magnification, ×100 or ×400; scale bar, 100 μm). (c). Cartilage OARIS scores. The data in the figure are mean ± SD. ***P < 0.01 by Student’s t-test (n = 5). (d) ELISA was performed to detect serum TNF-α, IL-1 β and IL-4 in mice. *P < 0.05, ***P < 0.01 by Student’s t-test (n = 5).

To evaluate the possible therapeutic effect of Sirt6 in the mouse OA model, we investigated the protective effects of Sirt6 on articular cartilage. A mouse surgical OA model was established by destabilization of the medial collateral ligament and medial meniscus, followed by tail vein injection of 10 μL of Lenti-Sirt6 or Lenti-NC at 0, 15, 30 and 45 days post-OA operation. At 8 weeks after surgery, immunoblot and qPCR were performed to quantitate Sirt6 amounts in synovial tissue of mouse knee joint. The results showed Sirt6 protein and mRNA amounts in synovial tissue were elevated in the Ad-Sirt6+ OA group compared with the Ad-LacZ+OA group (Figure 5a). Histological assessment of OA was performed by H&E and Safranin O–fast green (S–O) staining of knee joint cartilage specimens. Pathological assessment revealed that knee joint samples from Ad-LacZ+OA mice showed classical OA features, with extensive cartilage missing, SO staining loss and disorganized chondrocytes. By contrast, knee joints from Ad-Sirt6+ OA mice showed a remarkable improvement (Figure 5b). Meanwhile, OARSI scores were utilized for quantitation. OARSI scores were 1.20 ± 0.21 and 3.46 ± 0.36 in the Ad-Sirt6+ OA and Ad-LacZ+OA groups, respectively, with a significant difference between the two groups (Figure 5c, p<0.01). To verify the anti-inflammatory effect of Sirt6, we performed ELISA to detect the changes of serum pro-inflammatory (TNF-α, IL-1β) and anti-inflammatory (IL-4) factors in mice at 50 days post-OA surgery. Compared with the Ad-LacZ+OA group, the Ad-Sirt6+ OA group had increased levels of IL-4 and reduced amounts of TNF-α and IL-1β (Figure 5d). Jointly, these findings demonstrated that Sirt6 overexpression attenuated OA progression in the mouse model.

## 4. Discussion

Previously, basal SIRT6 gene expression in unstimulated articular chondrocytes derived from normal cartilage from adults aged 17–72 yrs was approximately 1.5 to 2 when lowest value was set to 1 and SIRT6 levels were normalized to YWHAZ as a housekeeping control [21]. In addition, SIRT6 activity is altered with age and oxidative stress conditions associated with aging [21]. This study showed that in comparison with acute meniscus injury patients, OA patients had decreased levels of Sirt6, the anti-inflammatory cytokine IL-4 and M2 macrophages, and elevated amounts of the pro-inflammatory cytokines TNF-α and IL-1β. We also demonstrated Sirt6 inhibition induced RAW264.7 cells to release pro-inflammatory cytokines *in vitro*. Furthermore, in order to evaluate the relationship between Sirt6 and macrophage phenotype, we stimulated RAW264.7 cells to polarize them into the M1 and M2 types. We found reduced Sirt6 amounts in RAW264.7 cells of the LPS + IFN-γ group compared with the PBS group, but higher amounts in the IL-4 group. We next demonstrated that Sirt6 inhibition could promote RAW264.7 cell polarization into M1 macrophages and inhibit their polarization into the M2 type. What’s more, Sirt6 overexpression alleviated OA and inhibited inflammation in mice. Therefore, effectively inducing Sirt6 might become a novel therapeutic approach in OA. Nevertheless, the mechanisms explaining the link between Sirt6 expression and synovial macrophage polarization still need further exploration.

Inflammation plays a critical role in OA development and progression. Increasing evidence shows that synovitis is related to the pathogenesis and development of OA [22,23]. The normal synovium has two different tissue layers. There are two or three layers of macrophages and fibroblast-like synoviocytes in the inner lining layer. The outer layer (subsynovial layer) comprises the fibrous connective tissue and blood vessels, with a limited number of lymphocytes or macrophages [24]. Different stimuli can lead to the polarization of macrophages into classically activated (M1) or alternatively activated (M2) macrophages [25]. The critical function of synovial macrophages in the pathogenesis of rheumatoid arthritis (RA) was recently demonstrated [26]. M1 macrophage polarization is induced through Notch, JNK and ERK1/2 signaling activation, leading to increased release of inflammatory molecules, including TNF-α, IL-1β, IL-6 and IL-23 in the development of RA [27]; meanwhile, M2 macrophages exert anti-inflammatory effects by releasing IL-10 and transforming growth factor-β (TGF-β) in RA [28]. However, the function of macrophage polarization in OA remains largely unknown. As shown above, M2 macrophages in OA synovial tissues were decreased compared with control synovial tissues. During the development of OA, macrophages aggregate and polarize in the synovium and articular cavity, suggesting a potential correlation between macrophages and OA [29]. Utomo and colleagues found that M1, rather than M2 macrophages, had significant effects on inflammation and degeneration in cartilage explants *in vitro* [30]. Recently, Dai and collaborators showed that squid type II collagen induces macrophage M2 polarization and promotes macrophage expression of pro-chondrogenic genes (TGF-β and IGF), which could significantly improve the microenvironment around chondrocytes and lead to the production of type II collagen and glycosaminoglycan to reduce OA [31].

In mammalians, sirtuins have seven isoforms (Sirt1-7). Sirtuins, which are evolutionarily conserved proteins showing NAD^+^-dependent deacylase activity, contribute to physiological and pathological processes, including inflammatory response, apoptosis, aging, metabolism and stress resistance [32,33]. It was recently demonstrated that depletion of Sirt6 can cause DNA damage, telomere dysfunction, and subsequent human chondrocyte senescence [34]. Knockout of the Sirt6 gene can increase the expression of pro-inflammatory cytokines (IL-1β, IL-6 and IL-8), the COX-prostaglandin system, and ECM remodeling enzymes (MMP2, MMP9 and PAI-1), which contribute to OA’s pathological process [35]. In addition, Sirt6 expression is significantly reduced in articular chondrocytes in clinical OA, and its overexpression prevents OA progression by alleviating chondrocyte aging and inflammation [18,19,34]. Furthermore, there is mounting evidence that improving Sirt6 activity may represent an effective strategy for OA treatment. Recent reports have confirmed cyanidin as an effective Sirt6 activator, which alleviates IL-1β-related ECM degradation and inflammation in human OA chondrocytes by regulating the Sirt6/NF-κB signaling axis [36]. Zhi et al. demonstrated that hydroxytyrosol inhibits inflammatory response in OA chondrocytes through Sirt6-dependent autophagy [37]. As mentioned above, Sirt6 can alleviate OA cartilage degeneration by inhibiting inflammation. In addition to arthritis, Sirt6 has significant anti-inflammatory properties in various inflammatory disease models, such as proteinosis, steatohepatitis and allergic airway inflammation [38]. It is known that synovitis has a critical function in OA progression. Recently, it was demonstrated that Sirt6 deletion facilitates pro-inflammatory M1 macrophages and augments the migration potential of macrophages toward adipose-derived chemo-attractants [39]. Woo and colleagues revealed that myeloid Sirt6 deficiency promotes the pathogenesis of rheumatoid arthritis by accelerating the migration of macrophages from circulation to joint tissues and favoring the M1 type 40. As shown above, Sirt6 expression was decreased in OA patients in comparison with control patients. Sirt6 can affect the polarization of synovial macrophages and the release of inflammatory factors both in cell and animal models. These findings reveal Sirt6 as a potential suppressor of OA development.

In summary, the current study demonstrated that Sirt6 represents an important mediator of OA progression. Sirt6 inhibition could promote the release of proinflammatory cytokines by macrophages in the synovial membrane, and induce M1 polarization and inhibit M2 polarization in macrophages. Moreover, overexpression of Sirt6 could alleviate OA and inhibit inflammation. Developing specific Sirt6 inducers may help prevent or treat osteoarthritis. It has been reported that SIRT6 can promote resistance to oxidative stress, which is one of the OA characteristics. Thus, we may focus on the role of SIRT6 in maintaining redox homeostasis in articular chondrocytes in the future.

## Data Availability

The datasets used during the present study are available from the corresponding author on reasonable request.
